# Does Adenotonsillectomy alter Symptoms of Attention Deficit Hyperactivity Disorder in Children?

**DOI:** 10.22038/ijorl.2020.43987.2456

**Published:** 2020-11

**Authors:** Reza Fallah, Aliasghar Arabi Mianroodi, Mahin Eslami, Narges Khanjani

**Affiliations:** 1 ***Department of Otolaryngology, Head and Neck Surgery, Shafa Hospital, Kerman University of Medical Sciences, Kerman, Iran.***; 2 ***Department of Psychiatry, Shahid Beheshti Hospital, Kerman University of Medical Sciences, Kerman, Iran.***; 3 ***Neurology Research Center, Kerman University of Medical Sciences, *** ***Kerman, *** ***Iran.***

**Keywords:** Adenotonsillar Hypertrophy, Attention Deficit, Hyperactivity, Tonsillectomy

## Abstract

**Introduction:**

Attention deficit hyperactivity disorder (ADHD) has the highest prevalence among psychiatric disorders in children. The present study investigated the effect of adenotonsillectomy on the symptoms of ADHD in a 6-month follow-up.

**Materials and Methods:**

This cross-sectional study was performed on 100 patients referred for respiratory problems during sleep due to adenotonsillar hypertrophy (ATH). The patients’ parents were asked to complete the Diagnostic and Statistical Manual of Mental Disorders Fourth Edition checklist as a standard benchmark for ADHD before, 2 weeks, and 6 months after the surgery. The data were analyzed by SPSS software (version 20) through paired t-tests and McNemar’s test.

**Results:**

The age averages of male and female children were 7.15 and 8.4 years, respectively. The frequency of ADHD in the studied population was 30%, which is much higher than the prevalence of this disorder in the normal population. In the second week after the surgery, the mean score of ADHD decreased from 4.97±2.97 (attention deficit [AD]) and 6.77±1.61 (hyperactivity disorder [HD]) before the surgery to 3.86±2.25 (AD) and 4.28±2.02 (HD) 2 weeks after the surgery (P=0.001). After a 6-month follow-up, these figures further decreased (AD=2.34±2.32; HD=1.97±2.44; P<0.001).

**Conclusion:**

Adenotonsillectomy had a significant effect on the improvement of ADHD symptoms. There is a necessity for checking patients with ADHD for ATH, especially in case of sleep disorders, sleep apnea, snoring, or mouth breathing.

## Introduction

Attention deficit hyperactivity disorder (ADHD) has the highest prevalence in psychiatric disorders among children and adolescents (1), as it contributes to 50% of referrals to psychiatric clinics (2-4). 

This disorder is a long-term illness manifesting itself throughout life, from preschool to puberty, and its manifestations may change from preschool to adulthood. The prevalence of this disorder is reported to be 3-7% in the Diagnostic and Statistical Manual of Mental Disorders Fourth Edition (DSM-IV) (5). The prevalence rates of ADHD in the United Kingdom, Colombia, and Bangkok were reported as 1% (6), 16.4% (7), and 6.5% (8), respectively. Several studies conducted in Iran indicated a relatively high prevalence of ADHD among school and preschool children. One of the aforementioned studies carried out by Alishahi et al reported the incidence of ADHD in school children in Shiraz, Iran, to be 5.8% (9). 

A limited number of studies have been conducted on the relation between adenotonsillar hypertrophy (ATH) and psychiatric and behavioral disorders. A study performed by Soylu et al. showed that in preschool children, in addition to mouth breathing and breathing disorders during sleep, ATH may also be associated with psychiatric symptoms and abnormalities in children within the age range of 5-12 years, and adenotonsillectomy improved the psychiatric symptoms and severity of these disorders in most cases (10). Passali et al. also reported that adenotonsillectomy can treat behavioral disorders (11). Adenoids are located, as parts of the lymphatic tissue, in the posterior region of the nasopharynx (12). Adenoid hypertrophy is a common health problem among children. The nasopharyngeal obstruction which is the result of adenoid hypertrophy can cause mouth breathing, snoring, sleep disturbances, poor nutritional status, sinusitis, and inflammation of the middle ear in children (13). The adenoid may act as a reservoir of pathogenic bacteria. An explanation for adenoid tissue removal is a chronic infection that may lead to adenoid hypertrophy (14). Sleep-disordered breathing is a relatively common problem and can be observed in about 1% of children (15). One reason for this problem in children is the hypertrophy of the adenoid and palatine tonsils. Sleep-disordered breathing symptoms include snoring, respiratory failure, difficulty in breathing, restless sleep, frequent awakening during night-time sleep, and behavioral disorders. In children suffering from sleep disorders, offensive behaviors, such as violence, learning disruption, and social withdrawal, are observed (15,16). Some researchers believe that obstructive sleep apnea may also cause problems in memory, comprehension, and daily activities (15).

Several studies have been carried out on the prevalence of ADHD in Iran. Ali Moradi et al. reported that the prevalence rate of ADHD in Neyshabur, Iran, was 12.5% (95% CI: 10-14.8%). The prevalence rates of ADHD were 10.9% (95% CI: 7.8-14%) and 14.3% (95% CI: 10.5-18.2%) in male and female adolescents, respectively. The prevalence rate of ADHD was higher in female subjects; however, the difference was not statistically significant (17).

In a study conducted by Ahmadi et al. in Besat Hospital in Hamedan, Iran, most children who were diagnosed with ADHD and underwent adenotonsillectomy improved within a month (18). With this background in mind, the present study was conducted to investigate the effect of adenotonsillectomy on the severity of symptoms of ADHD in children within the age range of 3-15 years referred for adenotonsillectomy to the ear, nose, and throat (ENT) clinic of Shafa Hospital in Kerman, Iran.

## Materials and Methods 

This cross-sectional study was carried out on a total of 100 children. The sample size was estimated based on a study by Ahmadi et al. (18). In this study, the mean scores of attention deficit (AD) before and one month after the intervention were 1.76±0.35 and 1.52±0.38, respectively. Moreover, by presuming a power of 0.9 and type I error of 0.05 and according to the equation for determining the sample size for studies comparing means, the minimum sample size need was 49 cases.

The sample size was also estimated based on the difference of the hyperactivity score; however, it resulted in a smaller sample size. The study subjects were selected from the patients referred to the ENT clinic of Shafa Hospital in Kerman within the age range of 3-15 years and undergoing adenotonsillectomy in 2014-2015. 

Convenience sampling was continued until 100 patients entered the study. The study objective was explained to the parents of the patients. In addition, written consent was obtained from the parents. The inclusion criteria were children with ATH who had sleep disorders, including snoring, apnea, restless sleep, and frequent awakening. 

These patients had a history of symptoms related to sleep breathing disorder and had frequently undergone antibiotic treatments; therefore, they had to go through surgical treatment as the final option. 

The exclusion criteria were other diseases causing upper airway obstruction (e.g., allergic rhinitis and nasal congestion), obesity, craniofacial anomalies, chronic inflammatory diseases, recurrent tonsillitis, acute tonsillitis, serious psychiatric illnesses (e.g., depression, psychosis, and mental retardation [i.e., intelligence quotient less than 70]), and major neurological disease, including seizure. 

All the demographic information, including age and gender, was recorded in a checklist. The presence of ATH was determined by physical examination and recorded in the checklist. 

The size of the tonsil and/or adenoid was graded from +1 to +4, as +1 and 4+ indicated 25% and 100% blockage of the airway, respectively. The magnitude of the adenoid blockage was based on the lateral radiography of the neck and amount of blockage in the choanae (19).The children’s parents were asked to complete the DSM-IV checklist under the supervision of a psychiatrist before, 2 weeks, and 6 months after adenotonsillectomy. This checklist includes 9 items for the diagnosis of AD and 9 items for the diagnosis of hyperactivity disorder (HD). In children under 16 years, the presence of at least 6 items for at least 6 months in each scale means the presence of AD or HD (20). The data were analyzed using SPSS software (version 20). The total score of the checklist was considered 9. The scores before and after adenotonsillectomy (2 weeks and 6 months) were compared by paired t-tests. The percentage of children with ADHD symptoms before and after the adenotonsillectomy was compared by the McNemar’s test. P-values of less than 0.05 were considered statistically significant.

## Results

A total of 100 children, including 53 male and 47 female subjects, entered this study, and all the children were followed up to the end of the study. The age averages of male and female children were 7.15 (range: 4-11 years) and 8.4 (range: 4-13 years) years, respectively. The frequency of ADHD in the studied population was 30%, which is much higher than the prevalence of this disorder in the normal population. Among the subjects with ADHD, 66% (n=20) and 33% (n=10) of the patients were male and female, respectively. The patients were assessed 2 weeks after the surgery, in which improvement was observed. After 6 months, more male and female children showed improvement in ADHD symptoms. (Table 1 and 2)

**Table 1:** Frequency of preoperative attention deficit hyperactivity disorder cases and attention deficit hyperactivity disorder in follow-ups

	**ADHD at baseline n (%)**	**ADHD after 2 weeks n (%)**	P-value*	**ADHD** ** after 6 months ** **n (%)**	P-value*
Male subjects (n=53)	20 (37.7)	11 (20.7)	0.001	6 (11.3)	<0.001
Female subjects (n=47)	10 (21.3)	5 (10.6)	<0.001	3 (6.4)	<0.001
Total (n=100)	30 (30.0)	16 (16.3)	<0.001	9 (9.0)	<0.001

ADHD: Attention deficit hyperactivity disorder

*McNemar’s test was used to compare the results to those reported before surgery.

**Table 2:** Mean scores of attention deficit and hyperactivity disorder before and after adenotonsillectomy based on the Diagnostic and Statistical Manual of Mental Disorders Fourth Edition

	Before surgery	2 weeks after surgery	P-value*	6 months after surgery	P-value*
AD score	4.967±2.967	3.862±2.248	0.001	2.345±2.319	<0.001
HD score	6.767±1.612	4.276±2.016	<0.001	1.966±2.442	<0.001

* Paired t-test was used to compare the results to those reported before surgery.

AD: Attention deficit , HD: Hyperactivity disorder

## Discussion

The ADHD is the most prevalent psychiatric disorder among children and adolescents (1) accounting for 50% of childhood psychiatric referrals (2-4). The present study investigated the effect of adenotonsillectomy on ADHD. According to the obtained findings, adenotonsillectomy had a significant effect on the reduction of ADHD in the children under study, which is in line with the results of previous studies in this regard. Amiri et al. also studied the effect of adenotonsillectomy on ADHD among 53 children in Tabriz, Iran. They reported that adenotonsillectomy significantly decreased the severity of ADHD symptoms 3 and 6 months following the operation. Similar to the results of the present study, the decrease was more significant at the 6-month postoperative evaluation, compared to that reported for the 3-month postoperative evaluation. Amiri et al. concluded that in children with sleep-disordered breathing associated with ADHD, adenotonsillectomy led to a significant decrease in the severity of ADHD symptoms (21). 

In another study conducted in Hamadan, Iran, 59 children within the age range of 6-12 years with ADHD underwent adenotonsillectomy, and ADHD symptoms improved in 41 and 51 patients 1 month and 3 months after the surgery, respectively. However, eight children were reported with no improvement. The AD and HD scores significantly decreased from 1.76 and 2.10 to 1.52 and 1.83 after 1 month and later to 1.24 and 1.52 after 3 months, respectively. The results of the aforementioned study showed that adenotonsillectomy is effective in the improvement of ADHD symptoms in children with ATH (18).

Dadgarnia et al. evaluated the effect of adenotonsillectomy on ADHD symptoms among 35 children within the age range of 5-12 years with ATH in Yazd, Iran. In the aforementioned study, 6 months after adenotonsillectomy, the frequency of the combined-type ADHD significantly decreased (54.3% versus 22.9%; P=0.003). The ADHD inattention score, HD score, and ADHD combined score significantly improved 6 months after the surgery. Dadgarnia et al. also concluded that upper airway obstruction occurring due to ATH is probably causing the symptoms of ADHD (22).

The results of a study conducted on 27 patients in Brazil showed that after tonsillectomy, daytime sleepiness, reaction time, and errors of omission and commission decreased. In addition, their performance in tests of visual attention improved (23). Similarly, Kim et al. conducted a study in Korea by enrolling children with ATH and sleep-disordered breathing. All the subjects underwent adenotonsillectomy, and the scores in all attention tasks, a year after treatment, significantly improved among these children. These authors also concluded that adenotonsillectomy can help improve attention in children with sleep-disordered breathing (24). In Turkey, a study was carried out on 41 patients within the age range of 6-11 years with a history of upper airway obstruction for whom adenotonsillectomy was performed. Based on the results, ADHD significantly decreased among the children after the operation (25). Another study conducted in Turkey on 64 children with an average age of 6.8 years showed that the mean of ADHD index, bedtime resistance, sleep-onset delay, sleep anxiety, night waking, parasomnias, sleep-disordered breathing, and daytime sleepiness significantly decreased after adenotonsillectomy (26).

Moreover, in the aforementioned study, it was suggested that physicians should check the symptoms of ATH among children with sleep disturbance and ADHD symptoms because adenotonsillectomy can improve the quality of life among these children (26).

Sleep-disordered breathing is common among 1% of children, and one of the possible causes is ATH (15,16). In a meta-analysis carried out by Ueno et al. in 2014, a statistically significant relation was shown between ADHD and sleep disturbance due to obstructive apnea caused by ATH (27). The main cause of ADHD is unknown; however, sleep disorders may play an important role in this regard, and this explains the reason for the improvement of the symptoms of these patients after tonsillectomy. In addition, the results of other studies demonstrated that tonsillectomy may significantly improve the results of the pulmonary function test in patients with large tonsils (28).

Many authors have suggested the assessment of children with ADHD symptoms for obstructive sleep-disordered breathing which can be caused by ATH (22,26). Sleep-disordered breathing in childhood may result in decreased arterial blood oxygen saturation and brain hypoxemia, thereby attributing to ADs in children (24). In a clinical trial conducted in multiple centers in the US on children within the age range of 5-9 years with obstructive sleep apnea without prolonged desaturation, the patients were randomly assigned to supportive care or early adenotonsillectomy. Nonverbal reasoning, fine motor skills, selective attention, and sleep significantly improved in the early adenotonsillectomy group (29). 

Other studies have reported that ATH causes increased arousal during sleep, shorter total sleep time, and decreased sleep efficiency in children (26). Scientists believe that most brain hormones that are related to neurodevelopment are secreted during night sleep; therefore, the quality of sleep can affect neurocognitive development (26). However, there are many factors causing ADs, and this explains why in a minority of children AD symptoms did not improve after adenotonsillectomy. One of the limitations of the present study was not enrolling controls, which could have been individuals with ATH and ADHD not undergoing the surgery; nevertheless, it was not possible due to ethical issues. Another limitation was that the patients were only followed up for 6 months. 

## Conclusion

According to the results of the present study, adenotonsillectomy had a significant effect on the reduction of the symptoms of ADHD among patients with ATH. Since ADHD is the most prevalent cause of the referral of children to psychiatric clinics, these patients should also be examined for ATH, especially if they have a positive history of sleep disorders, sleep apnea, night-time snoring, or mouth breathing.
